# Pentastomiasis

**DOI:** 10.4269/ajtmh.23-0275

**Published:** 2023-11-20

**Authors:** Pierre Marty, Coralie L’Ollivier, Loïc Simon

**Affiliations:** ^1^ Service de Parasitologie-Mycologie, Centre Hospitalier Universitaire de Nice, Nice, France;; ^2^ Université Côte d’Azur, INSERM 1065 C3M, Nice, France;; ^3^ Aix Marseille Université, Assistance Publique-Hôpitaux de Marseille, Institut de Recherche pour le Développement, Service de Santé des Armées, VITROME: Vecteurs–Infections Tropicales et Méditerranéennes, Marseille, France;; ^4^ Institut Hospitalo-Universitaire Méditerranée Infection, Marseille, France

**Figure 1.**
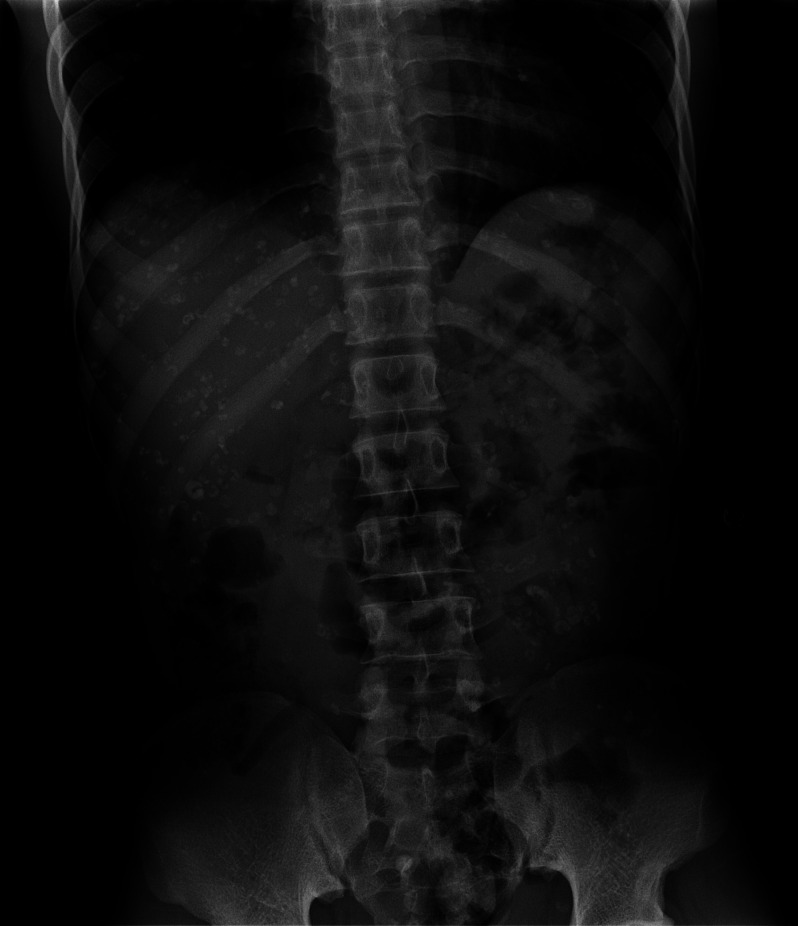
Abdominal X-ray of a young male patient showing a large number of horseshoe-shaped calcifications in the peritoneal cavity, indicative of pentastomiasis.

**Figure 2.**
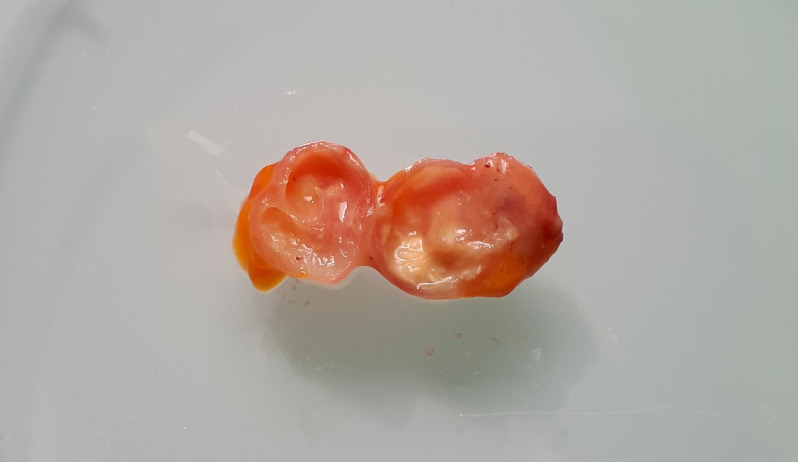
Macroscopic aspect of the biopsy collected during laparoscopy (length, 14 mm), showing a calcified cyst with internal aspect evoking a larval form.

A 21-year-old man from Guinea presented to the Teaching Hospital of Nice, France, with intestinal subocclusion. Hundreds of larva-like calcified forms were seen on X-ray and by an abdominal computed tomography scan inside the peritoneal cavity. The characteristic horseshoe shape of the calcifications (Figure 1) brought up the diagnosis of pentastomiasis. Exploratory laparoscopy showed no active larvae but biopsies of horseshoe-shaped calcifications were performed (Figure 2). DNA sequencing confirmed the presence of *Armillifer* sp., one of the parasites responsible for pentastomiasis in West Africa.^1^ *Armillifer* sp. adults inhabit the respiratory tract of specific snakes. Eggs are shed into the environment through the snake’s faeces and/or respiratory secretions. Humans can serve as a dead-end intermediate host by accidentally ingesting eggs from the environment or by eating undercooked infected snakes or handling them.^2^ Our patient explained to us that he had never eaten snake meat but that he had potentially been in contact with them in his village because some people were eating them. Therefore, he was probably infected by ingesting environmental eggs as a result of his proximity to snakes. Apart from effective pharmaceutical treatment that restored the intestinal transit (phloroglucinol, trimebutine, and metoclopramide), no specific action has been taken because the only known treatment would have been the mechanical removal of living larvae. When he was discharged from the hospital, the patient was doing very well.
